# *Drosophila* ML-DmD17-c3 cells respond robustly to Dpp and exhibit complex transcriptional feedback on BMP signaling components

**DOI:** 10.1186/s12861-019-0181-0

**Published:** 2019-01-22

**Authors:** Scott J. Neal, Darin Dolezal, Nisveta Jusić, Francesca Pignoni

**Affiliations:** 1 Center for Vision Research and Department of Ophthalmology, Upstate Medical University, NRB-4610, 505 Irving Ave, Syracuse, 13210 NY USA; 2 Department of Biochemistry and Molecular Biology, Upstate Medical University, Syracuse, NY USA; 3 Department of Neuroscience and Physiology, Upstate Medical University, Syracuse, NY USA; 4 Current Address: Department of Surgical Pathology, Yale-New Haven Hospital, New Haven, CT USA

**Keywords:** Bone morphogenetic protein (BMP), Decapentaplegic (Dpp), Schneider (S2) cells, ML-DmD17-c3 cells, Phospho-mad (pMad), Daughters against Dpp (Dad), Punt (Put), Thickveins (Tkv), Wishful thinking (Wit), Saxophone (Sax)

## Abstract

**Background:**

BMP signaling is involved in myriad metazoan developmental processes, and study of this pathway in *Drosophila* has contributed greatly to our understanding of its molecular and genetic mechanisms. These studies have benefited not only from *Drosophila*’s advanced genetic tools, but from complimentary in vitro culture systems. However, the commonly-used S2 cell line is not intrinsically sensitive to the major BMP ligand Dpp and must therefore be augmented with exogenous pathway components for most experiments.

**Results:**

Herein we identify and characterize the responses of *Drosophila* ML-DmD17-c3 cells, which are sensitive to Dpp stimulation and exhibit characteristic regulation of BMP target genes including *Dad* and *brk*. Dpp signaling in ML-DmD17-c3 cells is primarily mediated by the receptors Put and Tkv, with additional contributions from Wit and Sax. Furthermore, we report complex regulatory feedback on core pathway genes in this system.

**Conclusions:**

Native ML-DmD17-c3 cells exhibit robust transcriptional responses to BMP pathway induction. We propose that ML-DmD17-c3 cells are well-suited for future BMP pathway analyses.

**Electronic supplementary material:**

The online version of this article (10.1186/s12861-019-0181-0) contains supplementary material, which is available to authorized users.

## Background

The Bone Morphogenetic Protein (BMP) signaling pathway plays key roles in metazoan development and stem cell maintenance, at wound healing sites, and in myriad other contexts [1–3]. In *Drosophila* the BMP signaling cascade is less complex [4], whereas in mammals it features many specialized or redundant elements. Some of the pioneering work in discovering fundamental molecular and cellular mechanisms of BMP signaling has been conducted in the fly [5–7], and this continues to be an active area of research as new BMP signaling modulators are identified [8]. Thus, the simpler *Drosophila* system represents an ideal paradigm in which to elucidate mechanistic contributions of core BMP pathway components and modulators.

In *Drosophila* there are three BMP-like ligands encoded by the genes *decapentaplegic* (*dpp*) [6, 9, 10], *glass bottom boat* (*gbb*) [11, 12], and *screw* (*scw*) [13], of which Dpp is the best characterized and has been shown to play diverse developmental roles. Signaling initiates upon ligand binding to one of the constitutive Type II BMP receptors (Punt – Put, or Wishful Thinking – Wit) which in turn associates with, and transactivates, one of the Type I BMP receptors (Thickveins – Tkv, or Saxophone – Sax) [14, 15]. The activated receptor complex recruits and phosphorylates an intracellular signal transduction component, the receptor-regulated R-SMAD transcription factor Mad (Mothers against DPP) [16]. Phosphorylated Mad (pMad) associates with the co-SMAD Medea (Med) and together they translocate into the nucleus to regulate gene expression [17–20]. Among the transcriptional targets of Dpp signaling are genes encoding the inhibitory I-SMAD Dad (Daughters Against Dpp) [17, 21], and downstream mediators of the response to Dpp such as Bam (Bag of Marbles) [22], and Brk (Brinker) [23–25]. The simplicity of the cascade and the powers of genetic manipulation in *Drosophila* render the fruit fly a premier system for the study of fundamental aspects of BMP signaling in vivo.

The strength of the in vivo analyses in this animal model has been increased by in vitro experiments in cell culture that have investigated the pathway at a biochemical level using some of the earliest *Drosophila* cell lines, the Schneider (S2) line [9, 11, 16, 26–32], and Kc167 cells [33]. In particular, S2 cells have been invaluable in elucidating a variety of basic properties of BMP signal transduction, although they are not inherently responsive to Dpp. S2 cells are routinely augmented via supplementation of pathway components (e.g. constitutively-activated Tkv receptor or exogenous Mad transducer) to evaluate signaling activity [16, 28–32]. Furthermore, diverse S2 isolates with drastically different transcriptomes are in use throughout the community [34], making it difficult to reconcile published results pertaining to pathway activity and modulation.

In this study, we investigated several molecularly characterized *Drosophila* cell lines [34] to select one more suited to BMP pathway analysis. We found the ML-DmD17-c3 cell line [35] to be inherently responsive to the Dpp ligand across a wide range of concentrations. We demonstrate the respective contributions of the four BMP receptors to signaling, and examine the intricate transcriptional feedback that results from pathway activation in these cells. Absent any augmentation, ML-DmD17-c3 cells recapitulate key aspects of BMP signaling in vivo and therefore represent a valuable alternative tool for mechanistic studies of this essential signaling pathway.

## Results

### Identification of ML-DmD17-c3 cells and characterization of their responsiveness to Dpp stimulation

**Fig. 1:**
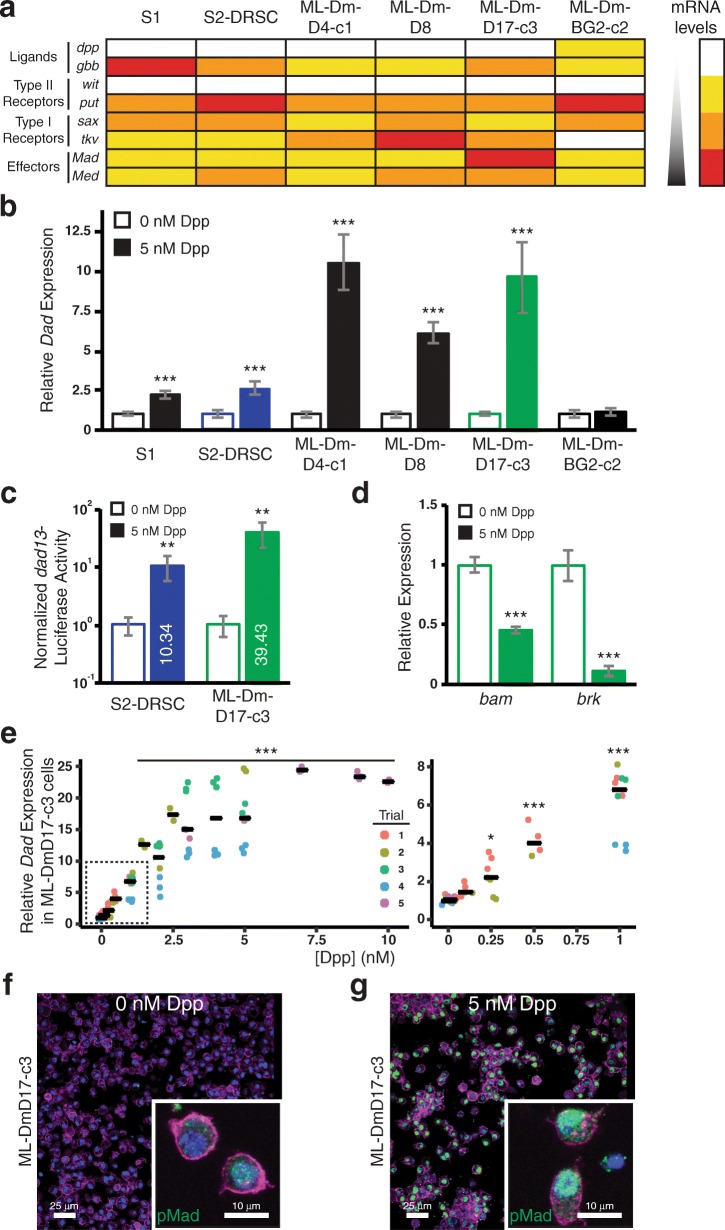
Identification of ML-DmD17-c3 (D17) cells, and characterization of their responsiveness to Dpp stimulation. (**a**) Graphical representation of gene expression values derived from modENCODE data [34] for each of six cell lines used in this study. The functional category and respective genes are listed to the left. Those with low (500–1000, yellow), medium (1000–2000, orange) and high (> 2000, red) expression are shaded proportionally to their expression values within each category. Expression values below 500 units are considered unreliable (white). It is only appropriate to compare expression values across cell lines within a gene, and not between genes (https://dgrc.bio.indiana.edu/cells/TilingDescription). (**b**) Quantification of relative *Dad* expression, normalized to *Act5C* expression, for each of the six cell lines used in this study, in the absence (empty bars) or presence (filled bars) of 5 nM recombinant Dpp. Baseline expression within each cell line was scaled to 1. Values given represent the mean and standard deviation of two independent assays, each with 2–3 technical replicates. *P* values were calculated for the pairwise comparison of means using Student’s t-test; *** *P* < 0.001. (**c**) Quantification of relative *dad13-*luciferase activity, normalized to *CMV-*Renilla activity, for S2 and D17 cells, in the absence (empty bars) or presence (filled bars) of 5 nM recombinant Dpp. Baseline expression was scaled to 1 for each cell line, and the fold-induction of *dad13*-luciferase activity is given within the filled bars; note the logarithmic axis. Values given represent the mean and standard deviation of two independent assays, each with 2–3 technical replicates. *P* values were calculated for the pairwise comparison of means using Student’s t-test; ** *P* < 0.01. (**d**) Quantification of relative *bam* and *brk* expression, normalized to *Act5C* expression, in D17 cells, in the absence (empty bars) or presence (filled bars) of 5 nM recombinant Dpp. Baseline expression was scaled to 1 for each gene. Values given represent the mean and standard deviation of two independent assays, each with 2–3 technical replicates. *P* values were calculated for the pairwise comparison of means using Student’s t-test; *** *P* < 0.001. (**e**) Quantification of relative *Dad* expression, normalized to *Act5C* expression, in D17 cells treated with the indicated concentrations of recombinant Dpp. Each assay is represented by a filled circle and independent assays are grouped by color, as indicated; median responses are indicated by black horizontal bars. The region contained within the dashed box is expanded to the right of the primary graph. Note that we observed larger variance between trials than within trials; we cannot explicitly account for these differences at this time. Data were analyzed using a general linear model using SPSS (IBM) and “Trial” was treated as a random factor. A *posthoc* Bonferroni test was used to calculate pairwise *P* values; * *P* < 0.05, ** *P* < 0.01, *** *P* < 0.001. (**f**, **g**) Representative images of immunocytochemical detection of pMad (green), cytoskeleton (magenta) and nuclei (blue) of untreated D17 cells (**f**) and of those treated with 5 nM recombinant Dpp (**g**) at low magnification and high magnification (insets)

Leveraging the transcriptome datasets produced by the modENCODE project [34, 36], we selected three candidate cells lines (ML-DmD4-c1; ML-DmD8; ML-DmD17-c3; [35]) with the highest transcript levels of key components of the Dpp signal transduction cascade (particularly *tkv*, *Mad*, and *Med*) (Fig. 1a, Additional file 1: Table S1). For comparison, we examined the established S2-DRSC (‘S2’ hereafter) and related S1 cell lines [26, 27, 34], as well as the central nervous system-derived ML-DmBG2-c2 cells [37]. Each cell culture was stimulated with 5 nM exogenous Dpp and transcript levels of the positively-regulated gene target *Dad* were measured by reverse transcription-quantitative (rt-q)PCR (Fig. 1b). ML-DmD4-c1 and ML-DmD17-c3 cells exhibited approximately 4-fold greater induction of *Dad* transcript than either S1 or S2 cells. Induction of *Dad* expression in ML-DmD8 reached an intermediate level, higher than in S2 but lower that in ML-DmD17-c3 cells. Lastly, expression of *Dad* was not affected by Dpp in ML-DmBG2-c2 cells; a result consistent with a failure to respond due to low expression of critical cascade components (Additional file 1: Table S1).

In particular, ML-DmD17-c3 cells (‘D17’ hereafter) displayed maximal relative *Dad* induction as well as the highest combination of *tkv*, *Mad* and *Med* expression [34]. To independently assess the robustness of this cell line’s response to Dpp, we measured pathway activation using a luciferase reporter driven by the minimal activating enhancer for the *Dad* locus [38]. We again observed increased pathway activation in D17 cells compared with S2 cells; a ~ 40-fold relative induction of luciferase activity in D17 cells stimulated with 5 nM Dpp as compared with a ~ 10-fold induction in S2 cells (Fig. 1c). Thus, D17 cells exhibited a similar magnitude of enhancement (4-fold) over S2 cells in the *Dad* response using the synthetic reporter construct as for endogenous transcript levels. Based on these observations we elected to further characterize the D17 cell line.

In addition to activating transcription, Dpp signaling acts via “silencer elements” to repress gene expression [39]. This has been extensively documented at the *bam* and *brk* loci [5, 39–42]. Hence, we sought to confirm this expected response to pathway induction in our experimental system. We found the expression of both genes to be consistently and robustly repressed by Dpp treatment in D17 cells (Fig. 1d).

Next, we explored the dose-response characteristics of D17 cells, as concentrations from 1 pM to 4 nM exogenous Dpp have been reported for experiments in S2 cells [29, 31, 32, 43, 44]. We tested Dpp concentrations from 0.1 nM to 10 nM and measured the *Dad* transcriptional response of D17 cells by rt-qPCR (Fig. 1e). We observed statistically significant *Dad* induction using as little as 0.25 nM Dpp and an essentially maximal response by 7 nM.

Lastly, Dpp pathway induction in S2 cells has been previously assessed by immunodetection of nuclear phosphorylated Mad (pMad) in cells [16, 31, 32, 45], and by Western blot detection of pMad from lysates of cells stimulated with as little as 10 pM Dpp [31]. Therefore, we sought to confirm that we could detect nuclear pMad accumulation in the D17 cell line by immunocytochemistry. Only sporadic D17 cells exhibited nuclear accumulation of pMad under the unstimulated condition (no Dpp) while those stimulated with 5 nM Dpp exhibited clear nuclear pMad accumulation (Fig. 1f-g).

In conclusion, the ensemble of experiments we have conducted demonstrate that native D17 cells exhibit all the hallmarks of robust BMP signaling.

### All four BMP receptors contribute to Dpp signaling in D17 cells

**Fig. 2:**
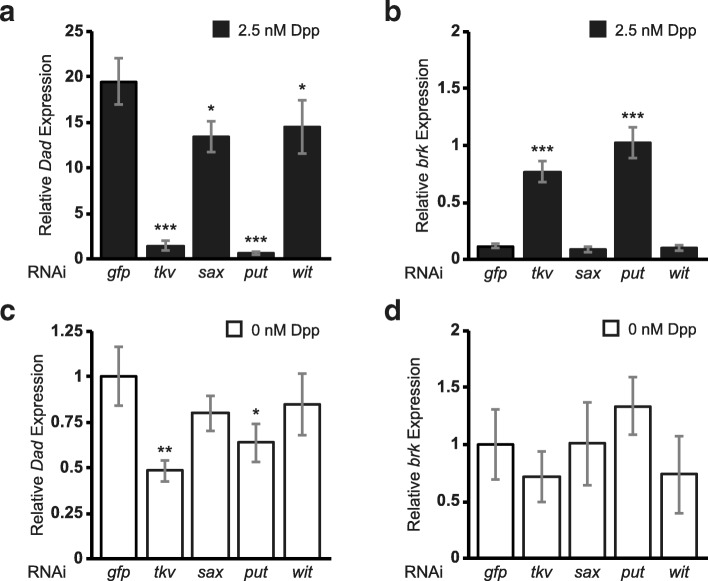
Put and Tkv are the primary transducers of Dpp signaling in ML-DmD17-c3 cells. Relative *Dad* (**a**, **c**) and *brk* (**b**, **d**) expression, normalized to *Act5C* expression, was measured in D17 cells treated with control (*gfp*) or receptor (*tkv, sax, put, wit*) RNAi, as indicated, in the presence (**a**, **b**; filled bars) and absence (**c, d**; open bars) of 2.5 nM recombinant Dpp. Values given represent the mean and standard deviation of two independent assays, each with 2–3 technical replicates. Data were analyzed using a general linear model in SPSS (IBM) and “Trial” was treated as a random factor. A *posthoc* Bonferroni test was used to calculate pairwise *P* values; * *P* < 0.05, ** *P* < 0.01, *** *P* < 0.001

To further examine the molecular mechanism of Dpp signal transduction in D17 cells, we assessed the contributions of the various Type I and II receptors. In S2 cells, Dpp-induced responses reflect the function of Tkv and Put, but not Sax [31]. Using the RNAi soaking method for *Drosophila* cultured cells [46, 47], we individually knocked-down (KD) each of the four receptor genes, *sax*, *tkv*, *put* and *wit* (Additional file 2: Figure S1a). Under Dpp-induced conditions, *put* or *tkv* KD almost-completely abrogated *Dad* induction as well as blocked *brk* repression **(**Fig. 2a-b). D17 cells were particularly sensitive to KD of either *put* or *tkv*; reducing transcript levels by as little as 20% resulted in a detectable decrease in maximal *Dad* induction (Additional file 2: Figure S1b-c). Furthermore, we observed a graded relationship between residual steady-state *put* or *tkv* transcript levels and Dpp-induced *Dad* transcriptional output (Additional file 2: Figure S1b-c). Maximal RNAi-mediated KD of *put* or *tkv* reduced the basal expression of *Dad* in unstimulated cells by 35 to 50%, respectively (Fig. 2c), but did not result in significant de-repression of *brk* (Fig. 2d). This observation suggests there is tonic BMP signaling in unstimulated D17 cells that contributes to the steady-state expression level of *Dad* but not *brk*.

Interestingly, KD of *sax* or *wit* yielded small yet significant reductions in *Dad* activation (by 25% and 30%, respectively), but had no effect on *brk* repression (Fig. 2a-b), thus revealing a differential response by these two gene targets, consistent with our observations in unstimulated cells. A priori, we did not expect to observe an effect of *wit* KD on pathway activity because modENCODE data showed *wit* expression to be in the low/unreliable range in D17 cells. However, as shown (Additional file 2: Figure S1d) and elaborated on below, Dpp-induction leads to enhanced expression of *wit* mRNA. Hence, the observed consequences of *wit* KD likely underscore a contribution of Wit to pathway activity.

In short, the transduction of the Dpp signal across the membrane of D17 cells is mediated primarily by the receptors Tkv and Put, with lesser contributions from Sax and Wit receptors.

### Transcription of pathway components is auto-regulated in Dpp-treated D17 cells

BMP signaling is subject to multiple levels of regulation [5, 48], which allow for it to function in diverse biological contexts. Autoregulation is essential in order to produce responses that are sequential or graded in space and time and, as mentioned in the introduction, two of the best characterized direct BMP pathway gene targets, *Dad* and *brk*, encode factors with autoregulatory activity [21, 25]. Dad functions as an I-SMAD, targeting Tkv for degradation [17, 21, 49]; Brk, a transcriptional repressor, competes with Mad for binding to certain gene regulatory sites [30, 38, 50]. Interestingly, the regulation of these factors in response to pathway induction is discordant. The strong upregulation of the signaling attenuator encoded by *Dad* contrasts with the reduced expression of the transcriptional repressor encoded by *brk*. Such “incoherent feedback” has been observed in several signaling pathways [43], and may be important for the capacity of a simple signaling cascade to generate diverse outputs [51, 52].

**Fig. 3:**
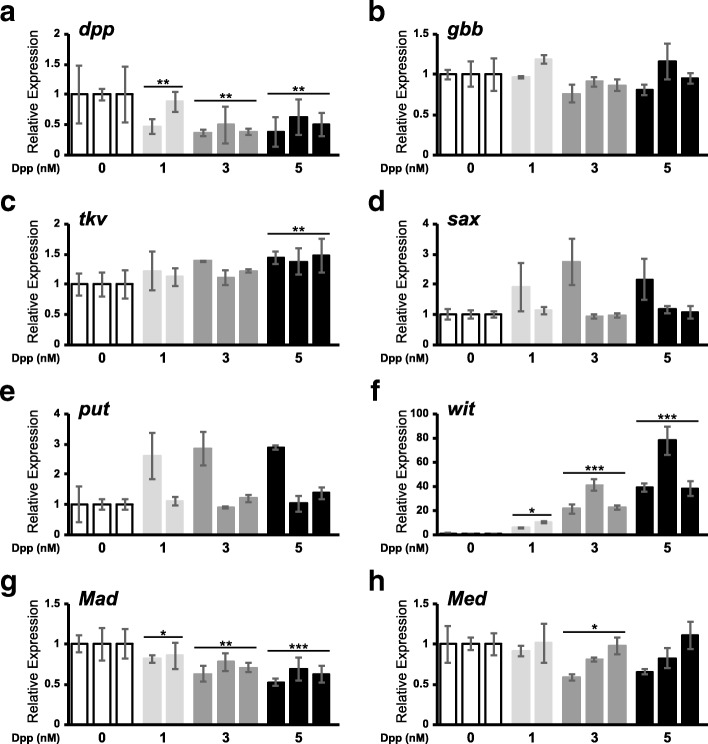
Transcription of pathway components is auto-regulated in Dpp-treated ML-DmD17-c3 cells. Relative expression of the indicated genes (**a**-**h**), normalized to *Act5C* expression, was measured in untreated cells (white bars) and in those treated with 1 nM, 3 nM or 5 nM recombinant Dpp (light gray, medium gray, and black, respectively). The Y-axes are scaled to the maximum expression for each gene. Each bar represents the mean and standard deviation of a single assay, consisting of 2–3 technical replicates. Baseline gene expression in untreated cells was normalized to 1 within each trial and the order of trials is maintained across treatments; “Trial 3” for 1 nM Dpp was not completed. Data were analyzed using a general linear model in SPSS (IBM) and “Trial” was treated as a random factor. A *posthoc* Bonferroni test was used to calculate pairwise *P* values; * *P* < 0.05, ** *P* < 0.01, *** *P* < 0.001

Thus, we explored the modulation of core pathway component expression as a potential additional level of feedback regulation. Specifically, we analyzed the mRNA level of the ligands *dpp* and *gbb* (Fig. 3a-b, respectively), the receptors *tkv*, *sax*, *put* and *wit* (Fig. 3c-f, respectively), and the intracellular transducers *Mad* and *Med* (Fig. 3g-h, respectively), in D17 cells treated with different concentrations of exogenous Dpp (1, 3, or 5 nM). We observed a significant reduction of *dpp* expression in all treatment conditions (*P*_Dpp_ = 0.037, *P*_Trial_ = 0.015, *P*_Dpp*Trial_ = 0.369) (Fig. 3a), while the mRNA level of *gbb* was unaffected (Fig. 3b). With respect to the receptors, we observed a small but significant Dpp-dependent increase in the expression of *tkv* in cells stimulated with 5 nM Dpp (*P*_Dpp_ = 0.036, *P*_Trial_ < 0.001, *P*_Dpp*Trial_ = 0.426; Fig. 3c). Small effects on *sax* and *put* transcripts were observed in only one of the three trials (Fig. 3d, e), whereas *wit* mRNA levels were consistently and dramatically upregulated in a Dpp dose-dependent manner (*P*_Dpp_ = 0.001, *P*_Trial_ = 0.179, *P*_Dpp*Trial_ = 0.002; Fig. 3f). Downstream of the receptors, the expression of *Mad* was downregulated by each concentration of Dpp tested (*P*_Dpp_ < 0.001, *P*_Trial_ < 0.001, *P*_Dpp*Trial_ = 0.965; Fig. 3g), whereas that of *Med* showed only slight repression with 3 nM Dpp (*P*_Dpp_ = 0.144, *P*_Trial_ = 0.022, *P*_Dpp*Trial_ = 0.132; Fig. 3h).

In summary, D17 cells modulate the expression of genes encoding BMP ligand (*dpp*), receptors (*tkv*, *wit*) and transducer (*Mad*) in response to BMP pathway activation by exogenous Dpp. The lowering of *dpp* and *Mad* mRNAs is anticipated to decrease pathway activity (negative feedback), whereas the dramatic increase in *wit* mRNA could reflect a compensatory loop that serves to sustain pathway activity in some contexts. Regardless, these effects suggest that complex mechanisms, beyond the direct regulation of factors such as *Dad* and *brk*, are at work to tailor BMP pathway activity, even within a simple cell culture system.

## Discussion

We have characterized the Dpp response of D17 cells and have shown that they exhibit robust and diverse transcriptional responses to exogenous Dpp stimulation. These effects are primarily mediated by Put and Tkv, with additional contributions from Sax and Wit, and result in feedback-regulation of core pathway genes — *dpp, tkv, wit* and *Mad*. In addition, these cells are amenable to the passive RNAi soaking method [46, 47, 53], facilitating RNAi-based approaches. Thus, D17 cells offer a paradigm that is uncomplicated by potential artifacts and limitations associated with manipulating pathway component levels by transfection. We believe that D17 cells are well-suited to analyses of constitutive and nuanced contributions of known and novel BMP pathway components to signaling output.

D17 cells were isolated from haltere imaginal discs [35], and exhibit a transcriptome consistent with the haltere hinge region [34]; the hinge/notum boundary is a region in the closely-related wing imaginal disc where Dpp signaling is essential [54, 55]. More recently it has been proposed that D17 cells exhibit haemocyte-like properties, including motility and phagocytosis [56]. However, D17 cells exhibit low *dpp* expression, unlike haemocytes [57, 58], and also form cell-cell junctions [56]. Origin aside, the results herein demonstrate that D17 cells are a versatile system for the study of BMP signaling.

In vivo, Tkv and Put play the major role in Dpp-initiated signaling, but different contexts and ligands also provide evidence of BMP signaling through the other receptors, Sax and Wit [59–63]. Tkv and Sax show partial functional overlap as well as distinct phenotypes [59]; in certain contexts, double-mutant combinations of *tkv* and *sax* display more severe phenotypes that more closely resemble *dpp* loss of function [59]. In the embryo, it was suggested that Sax may respond to only high levels of Dpp whereas Tkv functions at lower Dpp ligand levels [64]. Alternatively, within the context of a gradient of BMP signaling in the wing, it has been suggested that constitutive Gbb/Sax signaling serves to enhance Dpp/Tkv signaling where the latter is at low levels [61]. The contributions of Sax and Wit to BMP signal transduction at different ligand concentrations could be explored using the D17 cell model system. Moreover, Sax has been shown to respond to the Gbb and Scw ligands in some biological processes, including wing development and embryonic ectoderm development, respectively [61, 65, 66], and D17 cells could also be used to assess the interplay among these ligands and receptors. Overall, these results suggest that the combined action of Tkv- and Sax-containing BMP receptors is necessary to fully decode the Dpp signal in vivo. In addition, Sax- and Wit- containing receptor complexes play principal roles in responding to the Scw and Gbb ligands, respectively [62, 65, 67, 68].

In response to Dpp stimulation, we observed decreased expression of *dpp*, although *dpp* expression was low to begin with. Dpp-dependent *dpp* regulation has been observed in multiple instances in vivo [61, 69, 70], and in vitro [43], yet the context ultimately determines whether these effects are positive or negative. In vivo, elevated expression of Tkv and constitutively-active Tkv were shown to downregulate *dpp* expression [61, 69], a paradigm likely mimicked by long-term (18–24 h) exposure of cells to exogenous Dpp (this study). On the contrary, short-term (30–60 min) exposure of S2 cells to Dpp resulted in increased *dpp* expression [43]; in vivo, *dpp* induction was also observed when Dpp was ectopically expressed in the anterior of developing eye imaginal disc [70]. Hypotheses regarding the temporal and mechanistic control of *dpp* expression, such as evaluating direct (pMad-dependent) versus indirect (secondary) BMP signaling effects, could be further explored in D17 cells.

In regards to the regulation of *Mad* and *wit*, our results raise the possibility of additional levels of pathway autoregulation. For instance, a recent report has shown that *wit* expression is indirectly regulated through the relief of Brk repression [71]. Investigating the transcriptional (or post-transcriptional) mechanisms for the observed changes in *Mad* and *wit* mRNA levels, and identifying relevant in vivo contexts for this regulation, represent avenues for future research. Altogether, these effects are predicted to both enhance (increased *wit*) and repress (decreased *dpp* and *Mad*) BMP signaling, providing additional evidence of incoherent feedback, as discussed above for *Dad* and *brk*. D17 cells may be particularly useful in dissecting how such discordant inputs are integrated to set a specific level of BMP signaling.

Several outstanding questions regarding the integrated response of cells to stimulation by BMPs remain unanswered. For instance, BMP receptors are thought to exist as heterotetramers [1], and little is known of how the composition of such complexes affects signal transduction, specifically as it pertains to that of distinct ligand heterodimers [11, 61, 68]. In addition, we have demonstrated that depleting specific BMP receptors results in differential effects on known direct transcriptional targets (*Dad*, *brk*). Given the evidence of activity for each of the four receptors in D17 cells, it may be possible to investigate the intricacies of BMP receptor interplay using this system. Such an analysis could benefit from examining a more comprehensive set of target genes and by using a more parallel approach, for example Nanostring technology [43].

One potential advantage of D17 cells in studying BMP signaling that we have not explored is contact-dependent signaling [72, 73], since unlike S2 cells, D17 cells form aggregates with cell-cell contacts [56]. Moreover, the extracellular milieu is a critical factor in the range and efficiency of Dpp signaling [74, 75]. D17 cells exhibit differential expression of several extracellular modulators of BMP signaling, relative to S2 cells [34] (Additional file 3: Table S2). D17 cells express higher levels of *dally* and *dlp* which encode heparan sulfate proteoglycans that increase the stability of Dpp and facilitate its transmission in the extracellular environment [72, 76]. Conversely, collagen IV (encoded by *vkg* and *Cg25C*) sequesters Dpp in the ovary and reduces its effectiveness [77]; transcript levels of both *vkg* and *Cg25C* are much lower in D17 cells compared with S2 cells. Finally, a family of related proteins encoded by *tsg*, *sog* and *cv-d* interact with BMP ligands to shuttle them across long extracellular distances, yet limit local ligand activity [29, 63, 78]; *cv-d* and *sog* have lower expression in D17 cells whereas *tsg* has low expression in both D17 and S2 cells. Determining which, if any, of these factors contribute to the robust Dpp response we have observed in D17 cells represents an avenue for future research.

## Conclusions

BMP signaling in the publicly available and molecularly characterized D17 cell line is robust. Dpp signal transduction, via contributions from all four BMP receptors, results in the nuclear accumulation of pMad and the transcriptional regulation of known direct target genes such as *Dad* and *brk*. Furthermore, we observed transcriptional regulation of genes encoding elements from multiple levels of the BMP signal transduction cascade. In conclusion, we believe that D17 cells offer a naturally responsive, simple, and well-defined in vitro system in which to comprehensively study unresolved aspects of BMP signaling.

## Methods

### Cell culture

*Drosophila* ML-DmD4-c1, ML-DmD8, ML-DmD17-c3 (D17), ML-DmBG2-c2, S1 and S2-DRSC (S2) cells [27, 35, 37] were purchased from the *Drosophila* Genomics Resource Center (DGRC stocks 126, 92, 107, 53, 9 and 181, respectively). Cells were grown at 25°-28 °C in M3 Medium (Sigma) supplemented with 10% heat-inactivated fetal bovine serum (SH30070.02, HyClone), 50 units/mL penicillin G + 50 μg/mL streptomycin sulfate (Gibco) and with (ML-DmD4-c1, ML-DmD8, D17, and ML-DmBG2-c2) or without (S1 and S2) 10 μg/mL human insulin (Sigma). All experiments were performed between cell passages 3–30.

### DNA constructs and dsRNA production

The “*dad13*” element [38] was amplified by PCR (Primers: Additional file 4: Table S3) and cloned into the luciferase expression vector pGL3 (Promega). This construct is similar in concept, but not identical, to that reported elsewhere [79]. The constitutive CMV-*Renilla* luciferase construct was used as a transfection control (Promega). For dsRNA production, gene specific DNA sequences were amplified from cDNA by PCR using T7 promoter-containing primers (Additional file 4: Table S3) and cloned into pGEM-T Easy (Promega) for re-amplification. dsRNAs were generated from 1 μg PCR template using the MEGAscript T7 kit (Ambion), following the manufacturer’s instructions. RNA was pelleted by centrifugation at > 18,000 x g at 4 °C for 15 min and re-suspended in 100–200 μL of nuclease free water (Invitrogen). Nucleotides 1–435 of the GFP coding sequence were used for production of negative control dsRNA.

### RNAi and Dpp treatments

Cells were plated at a density of 2 × 10^6^ cells/mL in 24-well plates (0.5 mL/well) and allowed to adhere to the bottom of the well (2 h). Growth medium was removed and serum-free M3 medium containing dsRNA (500 pg – 5 μg, as indicated) was exchanged daily, for 3 days. Knockdown of target genes was verified by rt-qPCR (Additional file 2: Figure S1a).

Two hours after the final dsRNA treatment the medium was replaced with complete M3 medium, with or without recombinant Dpp (159-DP-020, R&D Systems) and RNA extractions were performed 18–24 h later. Note that commercial Dpp is provided as a disulfide-bridged homodimer; however, throughout this manuscript we report the effective monomeric concentration.

### Luciferase assays

S2 and D17 cells were plated to a density of 2 × 10^6^ cells/mL in 12-well plates and incubated for 24 h. Transfections were performed using jetPRIME (Polyplus Transfection). After 24 h, fresh complete media, with or without recombinant Dpp, was added for an additional 24 h. Cells were lysed at room temperature and Firefly/*Renilla* luciferase activities were immediately assessed using the Dual Luciferase Reporter Assay System (Promega) and a TD-20/20 Luminometer (Turner Designs).

### RNA isolation and rt-qPCR

RNA was isolated using RNAzol RT (Molecular Research Center, Inc.) and 1 μg total RNA was reverse-transcribed using oligo(dT)_20_ and SuperScript III (Invitrogen) under standard conditions. Quantitative PCR on 10 ng cDNA was performed using LightCycler 480 SYBR Green I Master Mix (Roche) according to the manufacturer, and a Bio-Rad CFX 384 Real Time PCR System. Cycling and melt-curve parameters were as follows: 95 °C 5 min; 45 cycles of 95 °C 15 s, 60 °C 15 s, 72 °C, 15 s; 70 cycles of 60 °C 5 s + 0.5 °C/cycle. Signals were recorded during the 72 °C extension phase and at each temperature during the melt analysis. Gene-specific amplification primers are listed in Additional file 4: Table S3. Melting curve analysis was used to confirm that each primer pair produced a single amplicon. The expression of *Actin 5C* (*Act5C*) was used as a “housekeeping gene” to normalize expression of genes of interest among samples and treatments. Normalized threshold cycle values were exported to Microsoft Excel and a custom analysis template was used to linearly scale baseline actin expression of control samples among qPCR runs in cases where data were pooled to produce a given figure.

### Immunocytochemistry, microscopy and image analysis

Treated and untreated cells were plated on poly-L-lysine (0.01%) coated coverslips in 6-well plates and allowed to settle (2 h). Cells were fixed (4% paraformaldehyde in phosphate-buffered saline (PBS) solution), washed (3X) in PBS and permeabilized and blocked in PBS containing 0.1% Triton X-100 (ThermoFisher) and 5% normal goat serum (G6767, Sigma) for 30 min before 1 h incubation in primary antibody solution. Primary antibodies used in this study were Rabbit-anti-phospho-Smad1/5 (1:250, Cell Signaling) [45] and Mouse-anti-DLG (1:200, Developmental Studies Hybridoma Bank). Cells were washed (2X) in PBS and re-blocked for 15 min, prior to the addition of fluorescently-conjugated secondary antibodies diluted in fresh blocking solution, supplemented with Alexa-546-conjugated phalloidin (1:50; ThermoFisher), for 1 h. Secondary antibodies used were Cy5-goat-anti-mouse IgG and Cy2-goat-anti-rabbit IgG (1:250, Jackson ImmunoResearch). Cells were washed (2X) 5 min in PBS, (1X) 10 min in PBS containing 0.1% Triton X-100, and (3X) 5 min in PBS. Cells were mounted in 2.5% (*w*/*v*) *n*-propyl-gallate dissolved in PBS containing 65% (*v*/v) glycerol, supplemented with 1:10^6^ Hoechst 33342 (final concentration 10 pg/mL; Sigma) and the coverglass was adhered to a standard microscope slide and sealed with nail polish.

Images were collected with a Leica SPE II confocal system attached to a DM5500Q base using a 40X oil-immersion objective (NA 1.15) with 1.5 zoom factor; insets were collected using a 100X oil-immersion objective (NA 0.70). Laser powers were adjusted to maximize, but not saturate signals in the Dpp-treated samples and were kept constant across all fields (5/coverglass) and slides within an experiment. Representative images are shown and all image adjustments (Leica LASX, Adobe Photoshop) were applied uniformly to all images at a given magnification.

### Statistical analysis

Unless otherwise stated, cell culture experiments consisted of 2 or 3 technical replicates and each experiment was repeated 2 or 3 times. rt-qPCR reactions were performed in triplicate. The average *Act5C*-normalized expression values were collected for each biological sample and differences between group means were compared using a general linear model in SPSS (v25, IBM). Independent trials were treated as random factors. A *posthoc* Bonferroni comparison was used to determine pairwise *P* values. Simple pairwise comparisons of luciferase and rt-qPCR data to determine Dpp treatment effects were analyzed using Student’s *t*-test (Microsoft Excel) to compare the means of the treated and untreated samples.

## Additional files

Additional file 1: **Table S1.** modENCODE gene expression values for core BMP signaling molecules. This table contains the calculated expression values for the indicated genes as originally reported by Cherbas and colleagues (2011). (DOCX 14 kb)

Additional file 2: **Figure S1.** Validation and titration of RNAi reagents in ML-DmD17-c3 cells. This supplemental figure provides evidence of the efficacy of RNAi treatments in ML-DmD17-c3 cells. (AI 1170 kb)

Additional file 3: **Table S2.** modENCODE gene expression values for extracellular modulators of BMP signaling. This table contains the calculated expression values for the indicated genes as originally reported by Cherbas and colleagues (2011). (DOCX 13 kb)

Additional file 4: **Table S3.** List of oligonucleotide primers used in this study. Oligonucleotide sequences (5′ to 3′) used for the generation of dsRNA and for the assessment of transcript abundance by rt-qPCR. (DOCX 16 kb)

## Abbreviations

Act5C: Actin at 5C
Bam: Bag of Marbles
BMP: Bone Morphogenetic Protein
Brk: Brinker
Cg25C: Collagen at 25C
Cv-d: Crossveinless-d
D17 cells: ML-DmD17-c3 cells
Dad: Daughters against Dpp
DGRC: *Drosophila* Genomics Resource Center
Dlp: Dally-like protein
Dpp: Decapentaplegic
DRSC: *Drosophila* RNAi Screening Center
dsRNA: Double-stranded RNA
Gbb: Glass Bottom Boat
KD: Knock-down
Mad: Mothers against Dpp
Med: Medea
p-Mad: Phospho-Mad
Put: Punt
RNAi: RNA interference
rt-qPCR: Reverse transcription-quantitative polymerase chain reaction
S2 cells: Schneider line 2 cells
Sax: Saxophone
Scw: Screw
Sog: Short Gastrulation
Tkv: Thickveins
Tsg: Twisted Gastrulation
Vkg: Viking
Wit: Wishful thinking
