# Supplementary material for: Drosophila ML-DmD17-c3 cells respond robustly to Dpp and exhibit complex transcriptional feedback on BMP signaling components
Source: BMC Dev Biol. 2019 Jan 22;19:1. doi: 10.1186/s12861-019-0181-0 (PMC6341649; doi:10.1186/s12861-019-0181-0)
Supplement: Supplementary file 1 — Table S1. modENCODE gene expression values for core BMP signaling molecules. This table contains the calculated expression values for the indicated genes as originally reported by Cherbas and colleagues (2011). (DOCX 14 kb) [file 12861_2019_181_MOESM1_ESM.docx]

**Table S1**: modENCODE gene expression values for core BMP signaling molecules.

|  |  |  |  |  | **Gene Expression Values**^1^ **in Candidate Cell Lines**^2^ | | | | | |
| --- | --- | --- | --- | --- | --- | --- | --- | --- | --- | --- |
| **Function**^3^ | **Gene**^4^ | **CG** | **Gene Name** | **Symbol** | S1 | S2-DRSC | ML-DmD4-  c1 | ML-DmD8 | ML-DmD17-  c3 | ML-DmBG2-  c2 |
| ligand | FBgn0000490 | CG9885 | decapentaplegic | *dpp* | 66 | 201 | 154 | 52 | 139 | 621 |
| ligand | FBgn0024234 | CG5562 | glass bottom boat | *gbb* | 2775 | 1131 | 803 | 983 | 1579 | 760 |
| receptor | FBgn0024179 | CG10776 | wishful thinking | *wit* | 181 | 68 | 28 | 147 | 146 | 156 |
| receptor | FBgn0003169 | CG7904 | punt | *put* | 1338 | 2040 | 1285 | 1709 | 1289 | 602 |
| receptor | FBgn0003317 | CG1891 | saxophone | *sax* | 1033 | 1344 | 788 | 1027 | 921 | 1151 |
| receptor | FBgn0003716 | CG14026 | thickveins | *tkv* | 640 | 650 | 1300 | 3754 | 1201 | 273 |
| effector | FBgn0011648 | CG12399 | Mothers against dpp | *Mad* | 832 | 824 | 860 | 571 | 2005 | 544 |
| effector | FBgn0011655 | CG1775 | Medea | *Med* | 978 | 1515 | 740 | 1213 | 1306 | 726 |

^1^ Gene expression values were originally published by Cherbas and colleagues (2011) as part of the modENCODE project.

^2^ Cell lines were obtained from the *Drosophila* Genomics Resource Center and were originally described by Schneider (1972; S1, S2), Ui and colleagues (1987; ML-DmD4-c1, ML-DmD8 and ML-DmD17-c3) and Ui-tei and colleagues (1994, ML-DmBG2-c2).

^3^ Function of the encoded gene product in the context of the canonical BMP signaling cascade.

^4^ FlyBase gene identifier, as reported by Cherbas and colleagues (2011).
