# Supplementary material for: Drosophila ML-DmD17-c3 cells respond robustly to Dpp and exhibit complex transcriptional feedback on BMP signaling components
Source: BMC Dev Biol. 2019 Jan 22;19:1. doi: 10.1186/s12861-019-0181-0 (PMC6341649; doi:10.1186/s12861-019-0181-0)
Supplement: Supplementary file 3 — Table S2. modENCODE gene expression values for extracellular modulators of BMP signaling. This table contains the calculated expression values for the indicated genes as originally reported by Cherbas and colleagues (2011). (DOCX 13 kb) [file 12861_2019_181_MOESM3_ESM.docx]

**Table S2**: modENCODE gene expression values for extracellular modulators of BMP signaling.

|  |  | |  | |  | |  | | **Gene Expression Values**^1^ **in**  **Candidate Cell Lines**^2^ | | |  |
| --- | --- | --- | --- | --- | --- | --- | --- | --- | --- | --- | --- | --- |
| **Function**^3^ | | **Gene**^4^ | | **CG** | | **Gene Name** | | **Symbol** | | S2-DRSC | ML-DmD17-  c3 | |
| Extracellular modulator | | FBgn0011577 | | CG4974 | | division abnormally delayed | | *dally* | | 832 | 1570 | |
| Extracellular modulator | | FBgn0041604 | | CG32146 | | dally-like | | *dlp* | | 193 | 491 | |
| Extracellular modulator | | FBgn0016075 | | CG16858 | | viking | | *vkg* | | 13634 | 166 | |
| Extracellular modulator | | FBgn0000299 | | CG4145 | | Collagen type IV | | *Cg25C* | | 12458 | 708 | |
| Extracellular modulator | | FBgn0003865 | | CG1502 | | twisted gastrulation | | *tsg* | | 0 | 84 | |
| Extracellular modulator | | FBgn0003463 | | CG9224 | | short gastrulation | | *sog* | | 508 | 102 | |
| Extracellular modulator | | FBgn0051150 | | CG31150 | | crossveinless-d | | *cv-d* | | 6487 | 2355 | |

^1^ Gene expression values were originally published by Cherbas and colleagues (2011) as part of the modENCODE project.

^2^ Cell lines were obtained from the *Drosophila* Genomics Resource Center and were originally described by Schneider (1972; S2) and Ui and colleagues (1987; ML-DmD17-c3).

^3^ Function of the encoded gene product in the context of the canonical BMP signaling cascade.

^4^ FlyBase gene identifier, as reported by Cherbas and colleagues (2011).
