# Supplementary material for: Drosophila ML-DmD17-c3 cells respond robustly to Dpp and exhibit complex transcriptional feedback on BMP signaling components
Source: BMC Dev Biol. 2019 Jan 22;19:1. doi: 10.1186/s12861-019-0181-0 (PMC6341649; doi:10.1186/s12861-019-0181-0)
Supplement: Supplementary file 4 — Table S3. List of oligonucleotide primers used in this study. Oligonucleotide sequences (5′ to 3′) used for the generation of dsRNA and for the assessment of transcript abundance by rt-qPCR. (DOCX 16 kb) [file 12861_2019_181_MOESM4_ESM.docx]

**Table S3**: List of oligonucleotide primers used in this study.

| **Gene** | **Purpose** | **Forward Primer (5' > 3')** | **Reverse Primer (5' > 3')** | **Source**^1^ |
| --- | --- | --- | --- | --- |
| *Act5C* | qPCR | GGCGCAGAGCAAGCGTGGTA | GGGTGCCACACGCAGCTCAT | Nevil et al., 2017 |
| *Dad* | qPCR | ATGATAAGTGCCACCACCTTTA | GTGCTGACTGGAATCGTAAGT | Dolezal, 2015 |
| *bam* | qPCR | GGGAGGTCCGATCTATTGCG | CGATCAGAGCGGAGAGGAAC | DRSC-PB^2^ #PD70062 |
| *brk* | qPCR | TCACGCCCCACTTTAAGCTG | CCGGCGGTGAATGTTGTATTTC | DRSC-PB #PP33156 |
| *tkv* | qPCR | CTTTGGCTCCATCATCATCTCC | TTCCGAAAATCTCGTCGTGC | Shimmi and O'Connor, 2003 |
| *sax* | qPCR | CCGGATCAACTGCCCATGATC | CATGTCAGAGCCGATGAATCC | Shimmi and O'Connor, 2003 |
| *put* | qPCR | CCACGGCAGGGAAACATTCAC | GGTCTTTGATGCCGGGATCTC | Shimmi and O'Connor, 2003 |
| *wit* | qPCR | AGCGGCAAGTATGGAACTGT | CATGGTACAGCGTTCATCGT | This study |
| *dpp* | qPCR | GCCAACACAGTGCGAAGTT | CGCCTTCAGCTTCTCGTC | Guo et al., 2013 (Forward) |
| *gbb* | qPCR | GATCTGGGCTGGCATGACTG | ATTCTCGTCGTTCAGGTGGT | This study |
| *Mad* | qPCR | GATGAGTGCGTGTGAGTG | CTTAGCTCGCTGTCCATTTTC | DeRobertis Lab^3^ |
| *Med* | qPCR | ACACGCAGTCTATGCAACCA | CTTGAAGGTCTCGCCCACTT | This study |
| T7^4^ | promoter | TAATACGACTCACTATAGGGA |  |  |
| *gfp* | dsRNA | T7-ATGGTGAGCAAGGGCGAGG | T7-GTTGTACTCCAGCTTGTGCCC | Dolezal, 2015 (PhD Thesis) |
| *tkv* | dsRNA | T7-AAGCACATCGGCAGCAGAG | T7-TCAGCATAAACACGGACAGGG | Shimmi and O'Connor, 2003 |
| *sax* | dsRNA | T7-CTCAATGGCAAGGAGCTACCG | T7-CGAGCCCAGTGGATAGTAGTG | Shimmi and O'Connor, 2003 |
| *put* | dsRNA | T7-GAGACAACGGGCATCCTGCGC | T7-GCCGCAGGGCTTGCCTGGCTG | Shimmi and O'Connor, 2003 |
| *wit* | dsRNA | T7-GAAGCCAGTCGGAATGTACCAG | T7-GAACACTCCATCAAAGGCAAGG | DRSC26091^5^ |

^1^ Documented primer pairs from the literature were used, as indicated.

1. Nevil M, Bondra ER, Schulz KN, Kaplan T, Harrison MM: **Stable Binding of the Conserved Transcription Factor Grainy Head to its Target Genes Throughout Drosophila melanogaster Development.** *Genetics* 2017, **205:**605-620.
2. Dolezal D: **A Novel Transmembrane Protein, Lilipod, Enhanced BMP Signaling and Promotes Germline Stem Cell Self-Renewal in the Drosophila Ovary.** 2015. (Ph.D. Dissertation, Upstate Medical University)
3. Shimmi O, O'Connor MB: **Physical properties of Tld, Sog, Tsg and Dpp protein interactions are predicted to help create a sharp boundary in Bmp signals during dorsoventral patterning of the Drosophila embryo.** *Development* 2003, **130:**4673-4682.
4. Guo Z, Driver I, Ohlstein B: **Injury-induced BMP signaling negatively regulates Drosophila midgut homeostasis.** *J Cell Biol* 2013, **201:**945-961.

^2^ Drosophila RNAi Screening Center PrimerBank; https://www.flyrnai.org/cgi-bin/DRSC_primerbank.pl

^3^ Sander, V. (2009); http://www.hhmi.ucla.edu/derobertis/ (Protocols>Fly Protocols>Primers for quantitative RT-PCR)

^4^ The T7 promoter sequence was appended to the 5’ termini of primers used to produce dsRNA.

^5^ Drosophila RNAi Screening Center; https://fgr.hms.harvard.edu/fly-cell-based-rnai
